# The Role of Temperature in the Growth and Flowering of Geophytes

**DOI:** 10.3390/plants2040699

**Published:** 2013-11-01

**Authors:** Nadezda V. Khodorova, Michèle Boitel-Conti

**Affiliations:** Unité de Recherche EA 3900 BIOPI “Biologie des Plantes et Innovations”, UFR des Sciences, Ilôt des Poulies, Jules Verne University of Picardie, 33 rue St-Leu, Amiens 80039, France; E-Mail: michele.boitel@u-picardie.fr

**Keywords:** bulb, geophytes, temperature, flowering

## Abstract

Among several naturally occurring environmental factors, temperature is considered to play a predominant role in controlling proper growth and flowering in geophytes. Most of them require a “warm-cold-warm” sequence to complete their annual cycle. The temperature optima for flower meristem induction and the early stages of floral organogenesis vary between nine and 25 °C, followed, in the autumn, by a several-week period of lower temperature (4–9 °C), which enables stem elongation and anthesis. The absence of low temperature treatment leads to slow shoot growth in spring and severe flowering disorders. Numerous studies have shown that the effects of the temperature surrounding the underground organs during the autumn-winter period can lead to important physiological changes in plants, but the mechanism that underlies the relationship between cold treatment and growth is still unclear. In this mini-review, we describe experimental data concerning the temperature requirements for flower initiation and development, shoot elongation, aboveground growth and anthesis in bulbous plants. The physiological processes that occur during autumn-winter periods in bulbs (water status, hormonal balance, respiration, carbohydrate mobilization) and how these changes might provoke disorders in stem elongation and flowering are examined. A model describing the relationship between the cold requirement, auxin and gibberellin interactions and the growth response is proposed.

## 1. Introduction

The term “geophytes” usually refers to species with a very short aboveground growth period in spring, which survive through the winter period not only by seed, but also in the form of specialized underground storage organs [1,2]. Although the underground organs are classified as true bulbs, corms or tubers of different types (stem, root or hypocotyl), according to the species, a broader definition for the term “bulb” can be applied to all geophytes, whether they are bulbous, tuberous or herbaceous [1]. 

**Figure 1:**
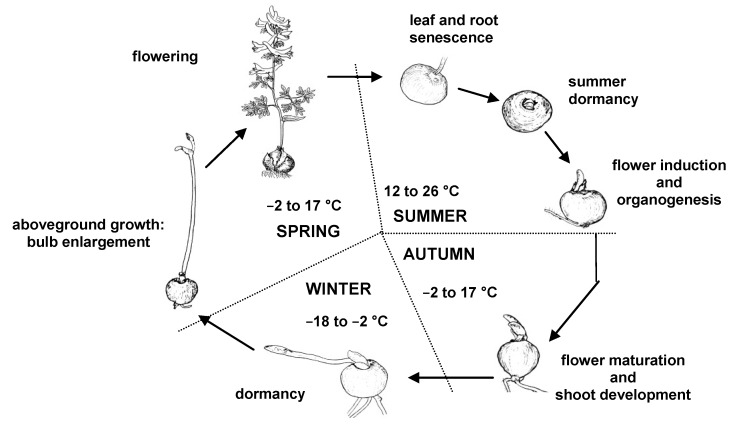
Schematic representation of the annual cycle of northern hemisphere geophytes using the example of *Corydalis solida* (adapted from [3]). The mean seasonal temperatures of a temperate continental climate are given.

Geophytes are characterized by a particular phenology (Figure 1). Plants that exhibit active growth and flowering during spring generally lose their aerial parts as summer begins. The senescence of aboveground tissues is followed by root senescence before the plant enters an apparently dormant period without visible organogenesis. During active aboveground growth, the carbohydrates accumulated due to photosynthesis are transported towards the underground organs, resulting in bulb enlargement. Bulb size is one of the major factors that determine the capacity for flowering. After a critical bulb size is reached, the flower meristem is induced and differentiated at the end of summer. Dormancy is broken in autumn, resulting in shoot and flower bud growth that continues throughout the winter. Therefore, for most of the year, these plants undergo an extended non-photosynthetic growth period.

Among several environmental factors (light, moisture, temperature) that can affect bulb development, temperature has been established as playing a predominant role in controlling growth and flowering in bulbs [1]. For most of them, a “warm-cold-warm” sequence is required to complete their lifecycle. Different genera and species demand various temperature optima, but, in general, the optimal temperature for the initial organogenesis ranges from 15 to 21 °С, while low positive temperature is required for the development of a flower stalk and well-formed flowers (Table 1) [1]. This period of autumn-winter cooling seems to be extremely important for flower development, as its absence leads to slow shoot growth and severe flowering disorders [4,5,6,7,8,9,10,11,12,13]. 

**Table 1:** Geographic origins of some ornamental geophytes and temperature requirements for their development [1,13,14].

Genera	Family	Origin	Temperature Requirements, ° C		
			Flower Induction and Organogenesis	Flower Maturation and Shoot Development	Aboveground Growth
*Allium*	Alliaceae	Temperate	9–13	5–8, during 16–24 weeks	8–11 for two weeks, then 17
*Hyacinthus*	Liliaceae	Irano-Turanian	17–25	4–9, during 10–18 weeks	10–13 for a couple of days, then 23
*Iris*	Iridaceae	Temperate	9–15	9–13, during 26–28 weeks	above 6
*Narcissus*	Liliaceae	Irano-Turanian	17–20	7–11, during 6–10 weeks	9–15
*Tulipa*	Liliaceae	Irano-Turanian	17–25	4–9, during 12–18 weeks	14–16

To date, relatively little is known about the physiological factors involved in stalk elongation and flowering, which are directly induced by low temperature. In this work, we review the currently known temperature requirements for flower initiation, development and anthesis in geophytes, with a particular emphasis on physiological mechanisms that are affected by temperature. A hypothesis of how temperature may regulate shoot elongation and flowering is presented. 

## 2. Temperature and Flower Induction and Initiation

Flower induction is a complex systemic process regulated by numerous genes, promoters and inhibitors, which are triggered by various signals, such as photoperiod and temperature [15,16]. The main research focuses on the determination of the optimal temperature for flower initiation and not on physiological studies of floral stimulus transition.

In most geophytes, the most important factor controlling their flowering is the seasonal thermoperiodicity, whereas the effects of light on flower induction are commonly less important. Most species do not show a preference for photoperiod, although in a small number of bulbous plants that respond to both temperature and day length (e.g., *Allium*, *Colchicum*), flowering is generally favored by short days [17,18]. 

In different species, flower induction and initiation may take place at different times of the year. For those in which these processes begin during the summer months (e.g., *Tulipa*, *Narcissus*, *Hyacinthus*, *Crocus*, some *Iris*), they are supported by relatively high temperatures. The temperature optimum for induction and early floral organogenesis also varies between species from 9 to 25 °C (Table 1). The physiological requirements are probably determined by the ecological origin of the species. For instance, those originating from temperate zones (e.g., *Allium*, *Iris*), usually require a very short period of relatively high temperature (about 20 °C) for flower induction, but this should be followed by a lower temperature for optimal flower meristem differentiation. Species originating from Irano-Turanian regions (e.g., *Tulipa*, *Narcissus*) require a relatively high temperature for flower induction, initiation and early differentiation (Table 1). The further the temperature is from the optimal range, the lower is the rate of floral meristem development. 

For all studied species, low temperature experienced in summer suppresses flower induction and initiation and leads to the formation of vegetative meristem [1,14,19,20,21].

High temperature (above 25–30 °C) experienced during the period when flower induction and initiation normally occur also delays them, but under these conditions, the flowering capacity of plants is maintained. The subsequent transfer to a lower temperature tends to favor the induction and organogenesis of flower buds [1]. 

Once flower differentiation is completed, most bulbs require a period of low temperature (4–9 °C) from the beginning of autumn, which enables stem elongation and anthesis in spring.

## 3. Physiological Changes during Flower Maturation and Shoot Development Affected by Temperature

Despite the apparent dormant state of bulbs during the autumn and winter months, active developmental processes continue to take place. All these processes require energy, carbohydrate partitioning and water uptake, which can only be supplied by the underground organ source [1,13]. The mobilization and transport of the reserves in bulbs seem to be affected by temperature conditions [1,13]. 

### 3.1. Water Status

During the storage period, growing buds are large recipients of water. Water redistribution in bulbs and its transfer to the buds plays an important role in the complete development of the flowers and is temperature-dependent. Indeed, in tulips, formed flower buds are aborted after storage in the wrong temperature conditions (17 °C). The water content in these flower buds is almost half of that in normal flowers of bulbs stored at optimal temperature (5 °C), thus suggesting that at least one of the possible reasons for bud abortion may be its insufficient water supply [9]. 

It has been reported that during storage, low temperature induces water transfer from lateral scales to central ones and also enhances the subsequent transfer of water from the basal plate and scales to the developing bud [7,8,9]. Water transport to the bud seems to be inhibited in some ways when the storage temperature is too high; however, the mechanism of this inhibition is still unclear. 

Moreover, low temperature storage leads to a selective expression of the aquaporin γTIP gene in stalks after planting [22]. Since aquaporins facilitate water transport during cell enlargement and contribute greatly to cell growth [23,24], the absence of γTIP gene expression might also play a role in water deficiency in buds and the inhibition of stalk growth in plants stored at ambient temperature. 

### 3.2. Respiration

Although flower bud development begins at a slow rate during the autumn-winter period, the growth of leaves and flowers takes place in spring. This active growth requires energy to supply, for example, ATP for enzyme working and sucrose loading to phloem. These energy demands might be provided by a temporarily increased respiration. 

Temperature does not appear to affect the respiratory activity of bulbs during storage, but, after planting, pre-cooled bulbs show significantly higher respiration and energy production than those stored at ambient temperature (about +18 °C), [25]. The respiration is higher both in the bulb scales and in developing shoots [26,27], probably due to the increased number of mitochondria following the cold treatment [28].

The respiratory metabolism is also affected by temperature during the epigenous growth of geophytes. Growing at warmer temperatures leads to a rapid increase in photosynthetic and photorespiratory rates, which results in faster starch accumulation and starch saturation in underground organs. The limit of the capacity for carbon accumulation in the sink organ provokes the feedback inhibition of the photosynthetic rate and, consequently, faster leaf senescence in comparison with plants growing at a lower temperature [29].

### 3.3. Carbohydrate Distribution

Among the physiological processes that occur in bulbs during storage that might be affected by temperature, most interest has been focused on the carbohydrate reserve, especially on its mobilization and further transport. 

It is well known that low temperature leads to the intensive activity of starch hydrolysis and the sucrose synthetic pathway [30,31], although it seems that starch hydrolysis in tulips and *Corydalis* is not influenced by temperature conditions during storage. Starch degradation also occurs in peripheral scales more intensively than in central ones, independently of storage temperature [8,10,11].

Since geophytes do not photosynthesize during the autumn-winter period, starch hydrolysis is the only source of soluble sugars. Thus, the dynamics of sugar accumulation in bulbs generally follow those of starch degradation. During storage, the total sugar concentration in bulbs is not influenced by differences in the surrounding temperature [11], and the amount of sucrose is always greater than that of reducing sugars [32]. The activities of sucrose-cleaving enzymes, sucrose synthase and the invertases remain at low levels [22]. 

However, even if the accumulation of sugars during storage is unlikely to be dependent on temperature, cold treatment has a considerable influence on sugar transport. For example, in tulip and *Corydalis* bulbs, low temperature storage leads to a transfer of nutrients from the storage organ to the bud, which is suppressed under a higher temperature regime [11,12,33]. 

For *Corydalis* plants, we have suggested a possible explanation for this transport inhibition based on the study of developmental and carbohydrate changes in tubers growing under natural autumn and winter temperatures (from 10 °C to −10 °C) and in tubers cultivated during the same period in a greenhouse at 18 °C [11,12]. We have shown that low temperature activates the apoplastic route for sugar movement from the storage parenchyma cells into the apoplastic region of the bulb. Apoplastic sugars are then loaded to phloem and transferred to the developing bud. The absence of low temperature prevents the efflux of sugars into the apoplast, thus blocking further delivery to the bud. However, the proposed hypothesis is based mainly on the particular ontogenesis of the *Corydalis* tuber and the absence of a cytoplasmic route for sugar loading in this species, thus it cannot be directly extrapolated to all bulbous plants.

Since the plants deprived of low temperature storage generally demonstrate only slight growth after planting, lower sugar concentrations in their bulbs and shoots are expected. Indeed, by the time of anthesis in tulip, the sugar content (hexoses, in particular) in stalks of correctly stored bulbs may be up to 10-fold higher than in bulbs stored at ambient temperature [5]. In fact, low temperature storage leads to an elevated expression of invertase genes and high activities of these enzymes in growing stalks, which maximizes hexose production [22,34,35]. 

There is no doubt that the accumulated carbohydrate reserve participates in shoot elongation. Since hexoses, particularly glucose, are known to contribute to cell division and elongation [36,37], it has been suggested that the slow growth of shoots in plants deprived of low temperature is due to an insufficient supply of sugars. Sucrose-cleaving enzymes (invertases and sucrose-synthase) are activated when there is a demand for metabolic carbohydrate and energy [38,39]. Apparently, without low temperature treatment, there is no induction of carbohydrate demand in shoots and no activation of invertases and sucrose-synthase [5,35]. At the same time, sucrose-cleaving enzymes play an important role in the control of plant development [40], and the low activities of these enzymes in the stalks of bulbs stored at higher temperature [34] may also prevent the initiation of stalk development and the subsequent storage of carbohydrates. 

## 4. Endogenous Plant Growth Regulators

The accumulations of endogenous abscisic acid and cytokinins in bulbs are not temperature-dependent processes [41,42], whereas the production of auxin and gibberellin is affected by the surrounding temperature and probably plays one of the leading roles in geophyte growth regulation. 

### 4.1. Gibberellins

Gibberellic acid (GA) is known for its role in the elongation of axial organs (stems, petioles and inflorescences), flower development [43,44] and vernalization [45]. 

For bulbous plants, it has been reported that the amount of GA correlates directly with shoot elongation rate and the presence/absence of cold treatment. After planting, when shoot elongation starts, the concentration of GA generally increases rapidly in the internodes and basal plates of pre-cooled plants, whereas non-cooled ones exhibit low GA levels in these organs [6,35,46,47]. 

Recently, it has been shown that *de novo* biosynthesis of GA is a prerequisite factor for shoot elongation in bulbous plants, which begins once shoot elongation has started. Biosynthesis of GA is also crucial for sprouting. The suppression of GA biosynthesis, even in cold-treated bulbs, leads to low stem growth [32,35]. 

### 4.2. Auxins

The role of auxins as factors involved in stem elongation and growth regulation in different plant species is widely discussed [48]. In bulbous plants, auxin is reported to be the main hormonal factor involved in the induction of tulip stalk elongation [47]. The primary site of auxin biosynthesis is the flower bud. The removal of floral buds causes weaker growth of the stalk, even in cooled bulbs, whereas the application of the exogenous auxin at the site of the removed buds induces normal elongation of the stem [1,49]. 

To date, auxin is the only known factor that shows a correlation between cold treatment requirement and the induction of stem and flower growth in bulbs. Rietveld *et al.* [47] have shown that, upon cooling, tulip stalks show a higher responsiveness and sensitivity to auxin, which increases gradually with the length of cold treatment. In the absence of cold treatment, plants are not sensitive to auxin signals. The authors suggested that this fluctuation in auxin response is the main factor responsible for the different growth behavior *in vivo* of cold-treated and untreated tulip bulbs. The response to auxin in stalks is not sufficient for their elongation without cold treatment [47]. 

It can also be mentioned that auxin is not only required for the induction of shoot elongation, but is also involved in it. Inhibition of auxin transport reduces stem elongation and results in a decrease in the quantity of gibberellins [49]. 

## 5. Are Auxin and Gibberellin the Main Agents in the Temperature-Dependent Growth of Geophytes?

**Figure 2:**
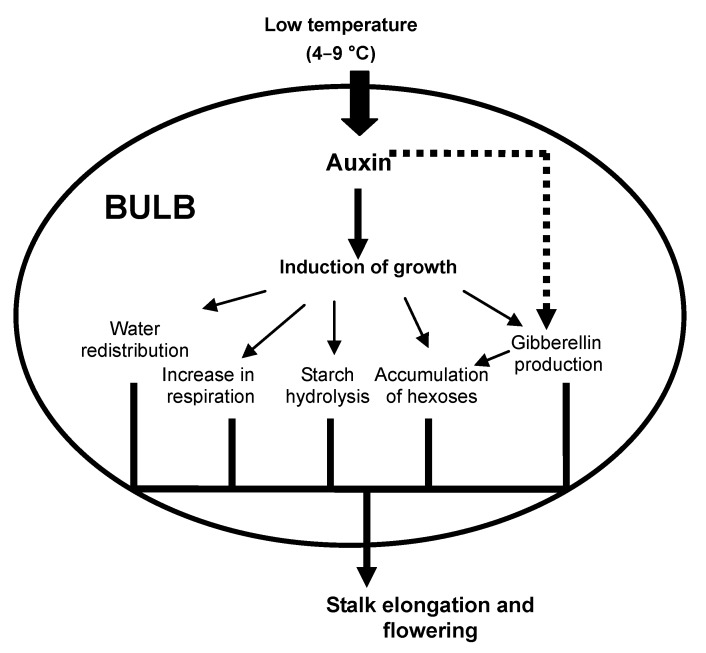
A proposed model of the influence of low temperature on growth in geophytes. Prolonged cold increases sensitivity to auxin, which induces shoot growth. The induction of growth triggers the remobilization of all reserves in bulbs, an increase in respiration and water flux and the onset of gibberellin biosynthesis. Accumulation of gibberellins leads to an enhanced expression of invertase genes, which provides the hexoses necessary for shoot elongation. All these parameters lead to proper stalk elongation and flowering. Auxin probably affects the onset of gibberellin synthesis (indicated by a dashed line).

The experimental data suggest that the only currently known cold-induced trigger in bulbs is auxin (Figure 2). In this case, temperature may act by changing the sensitivity of a plant to auxin signals and, thus, represents a growth-regulating factor.

In different plant species, both auxin and GA have central roles in temperature-controlled elongation responses [50]. Moreover, the concentration of auxin may regulate key genes encoding enzymes for GA biosynthesis [51,52,53,54,55]. This joint action of two hormones guarantees the growth of shoots and leaves: auxin promotes GA biosynthesis, thus acting as a mobile factor, whereas GA is the actual effector of growth [51,52]. 

Since stalk elongation in bulbous plants has also been shown to be due to the parallel activity of auxin and gibberellins, we suggest that auxin signals not only play a role in the induction of shoot growth, but also probably affect the onset of GA biosynthesis. The accumulation of the latter in growing shoots induces the remobilization of all reserves in bulbs in order to supply nutrients for growth. For instance, accumulation of GA leads to an enhanced expression of invertase genes, which establishes a large sink of sucrose transported from the bulb and provides hexoses necessary for shoot elongation. A normal growing shoot also induces an increase in respiration and water flux. 

Therefore, according to our hypothesis, the differences observed between pre-cooled and non-cooled plants during storage and after planting (in carbohydrate content, enzyme activities, water content and respiration rate, *etc.*) independently do not explain growth inhibition. These parameters are only secondary effects of the absence of growth, which is regulated by temperature via auxin signals and GA biosynthesis. 

## 6. Temperature and Aboveground Growth and Biomass Production

The effects of growth temperature on perennials are wide ranging, influencing many aspects of development, including leaf morphology, petiole length and stem thickness [56]. Early spring plants start their aboveground development under particular ecological conditions, *i.e*., comparatively low air and soil temperatures and high insolation. However, these parameters are not essential environmental factors for the completion of their short lifecycle in spring [1].

Once the leaf apex has emerged early in spring, the rapid growth of the whole plant starts. This could be guaranteed by the fact that the chloroplast structure is formed during underground growth in darkness, prior to the appearance of photosynthesis, which allows the plant to reach a maximum photosynthetic rate rapidly [57,58,59]. 

Relatively high temperatures (18/14 °C day/night temperature) during the growth period lead to faster growth in geophytes and earlier flowering. However, in this temperature range, the plants have smaller flowers and exhibit a shorter duration of vegetation. Moreover, a warmer spring always results in a lower bulb yield than for plants grown at lower temperature. In a cooler temperature (12/8 °C), bulb growth and leaf activity are maintained for a longer period [1,29,60,61,62]. Shorter growth time can also be explained by early activation of different sucrose-cleaving enzymes in the bulbs grown under warmer temperatures, which results in the faster shift from elongation to cell maturation and to the earlier cessation of starch accumulation [29,62]. Additionally, the rate of carbon assimilation at warmer temperatures rapidly becomes too high for the capacity to incorporate carbon into the bulb, leading to feedback inhibition of the photosynthetic rate.

Since the size of the bulb is considered to be one of the major parameters that influence the quality of flowers, low temperature conditions are more favorable during the aboveground growth of early spring plants. This will promote the production of larger underground organs and flowers in the next spring.

## 7. Conclusions

Almost all steps of growth and development in geophytes are controlled by seasonal thermoperiodicity. Although there have been many studies on temperature effects, which regulate the growth cycle of bulbous plants, the data obtained for different species are not directly comparable. We can certainly make inferences from concepts developed in different species concerning temperature-dependent growth. However, more thorough research of bulbous species is needed to provide considerable progress in understanding the correlation between seasonal thermoperiodicity and the annual growth cycle of geophytes. 
